# Long-term quality of life in adult craniopharyngioma patients: correlation with pituitary stalk section after endoscopic endonasal surgery

**DOI:** 10.1007/s00701-026-06902-7

**Published:** 2026-05-22

**Authors:** Irene Portonero, Enrico Lo Bue, Alessandro Pesaresi, Alice Antico, Silvia Sgambetterra, Raffaele De Marco, Federica Penner, Diego Garbossa, Michele M. R. Lanotte, Francesco Zenga

**Affiliations:** 1 Pituitary and Skull Base Surgery Unit, “Città Della Salute E Della Scienza” University Hospital, Corso Bramante 88, 10126 Turin, Italy; 2 Department of Neuroscience “Rita Levi Montalcini”, University of Turin, Turin, Italy

**Keywords:** Quality of life, Craniopharyngioma, Endoscopic endonasal approach, Pituitary stalk

## Abstract

**Purpose:**

Craniopharyngiomas are benign but locally aggressive tumors whose surgical treatment poses a challenge due to their proximity to critical structures. While gross total resection (GTR) may reduce recurrence risk, it often leads to significant long-term morbidity, especially when the pituitary stalk is sacrificed. This study aims to assess health-related quality of life (QoL) in adult patients undergoing endoscopic endonasal approach (EEA) for craniopharyngioma, with a focus on the impact of pituitary stalk section.

**Methods:**

We retrospectively analyzed 35 adult patients who underwent EEA for histologically confirmed craniopharyngioma between April 2012 and September 2023. Inclusion criteria were age > 18 years and at least one year of follow-up. Patients completed a custom 8-item telephone questionnaire assessing the impact of hormone replacement therapy (HRT), visual changes, hypothalamic dysfunction (sleep, appetite, sexual and behavioral disturbances), and nasal or olfactory symptoms. Each item was rated on a 1–5 scale, and patients identified the most burdensome symptom.

**Results:**

Older patients (> 55 years), those without preoperative panhypopituitarism, and those with postoperative hormonal decline reported higher impact. Visual deficits significantly affected QoL, particularly when vision worsened postoperatively. Obese patients (BMI > 30) reported greater impact from weight gain. Sleep disturbances were more prevalent in adamantinomatous craniopharyngiomas, while behavioral changes were more frequent in older patients. Smokers reported more nasal breathing issues. Sexual dysfunction was more frequently reported by males, older patients, and those who had stalk resection or new endocrine deficits. Stalk section was significantly associated with higher GTR rates but also with worsened endocrine function and higher cumulative QoL burden.

**Conclusions:**

Pituitary stalk resection increases GTR likelihood but at the cost of greater long-term endocrine morbidity. Patient age and baseline function significantly influence postoperative QoL, underscoring the importance of individualized surgical planning.

## Introduction

Craniopharyngiomas are complex tumors with a significant impact on patients' quality of life (QoL), both from surgical and endocrinological perspectives. Their location near critical structures such as the optic chiasm and pituitary stalk makes surgical management particularly challenging due to the risk of postoperative complications [17, 24, 32].

Effective treatment requires a multidisciplinary approach both before and after surgery. Endocrinologists assess hypothalamic-pituitary function and manage hormone replacement therapy as needed. Ophthalmologists evaluate visual field deficits often caused by optic pathway compression. When possible, collaboration with ENT surgeons is recommended to assist with harvesting the nasoseptal flap and managing postoperative care, including the treatment of cerebrospinal fluid (CSF) leaks. Neurosurgeons are responsible for selecting the surgical approach that best balances the extent of resection (EOR) with the risk of complications. Despite comprehensive evaluation and treatment planning, craniopharyngioma patients often experience significantly reduced QoL due to the effects of tumor growth and surgical intervention [7, 11, 18, 31]. After over a decade of experience managing these tumors, our team sought to evaluate the long-term impact of surgery on patients’ QoL.

In particular, the sectioning of the pituitary stalk has been associated with higher rates of gross total resection (GTR) but may also contribute to worsened long-term outcomes [17, 29, 40]. One of the main goals of this study was to assess how pituitary stalk sacrifice affects patients' QoL over time. To this end, we retrospectively analyzed a cohort of 35 patients who underwent endoscopic endonasal approach (EEA) for craniopharyngioma, with a specific focus on QoL outcomes and the factors most closely associated with postoperative impairment.

## Methods

This study is a retrospective single-center analysis of patients with craniopharyngioma who underwent endoscopic endonasal surgery between April 2012 and September 2023 at the authors' institution. Inclusion criteria were age over 18 years, histologically confirmed craniopharyngioma, treatment via EEA, and at least one year of follow-up. Exclusion criteria included alternative histological diagnoses and transcranial or transventricular surgical approaches.

All patients received preoperative endocrinological evaluation to assess hypothalamic-pituitary function and determine the need for hormone replacement therapy (HRT). Visual function was assessed preoperatively via computerized visual field testing. In urgent cases, endocrinological evaluation was performed postoperatively, and ophthalmologic assessment was deferred to three months after surgery.

Preoperative ENT evaluation was conducted to assess nasal anatomy. All patients underwent contrast-enhanced MRI with pituitary protocol to define tumor morphology and relationships with adjacent structures. CT angiography (CTA) was performed to evaluate vascular anatomy, including the relationship of the lesion with internal carotid and anterior cerebral arteries. MRI and CTA datasets were fused for intraoperative neuronavigation.

Postoperatively, copeptin levels and fluid balance were monitored every 12 h. Daily evaluations included serum electrolytes, blood glucose, plasma and urine osmolality to allow early diagnosis of central diabetes insipidus and guide Desmopressin therapy. On postoperative day 2 (POD-2), cortisol levels were measured to assess adrenal function. In patients already receiving corticosteroids, cortisol was not measured and Cortisone Acetate was continued prophylactically.

ENT follow-up was performed on POD-2 and POD-7 to assess the mucosal flap and detect bleeding or CSF leaks. Subsequent ENT evaluations were scheduled based on symptoms. Multidisciplinary follow-up (endocrinological and neurosurgical) was conducted one month after surgery, followed by neurosurgical assessments at six months and annually. Endocrine follow-up was tailored individually. Patients with preoperative visual deficits underwent ophthalmologic re-evaluation three months after surgery or earlier in case of visual worsening.

### Quality of life assessment

In all patients with at least one year of follow-up, QoL was assessed using a questionnaire specifically developed by the authors, as no validated instruments currently available in the literature comprehensively address the multiple QoL domains relevant to adult patients with craniopharyngiomas treated via an endoscopic endonasal approach.

**Fig. 1:**
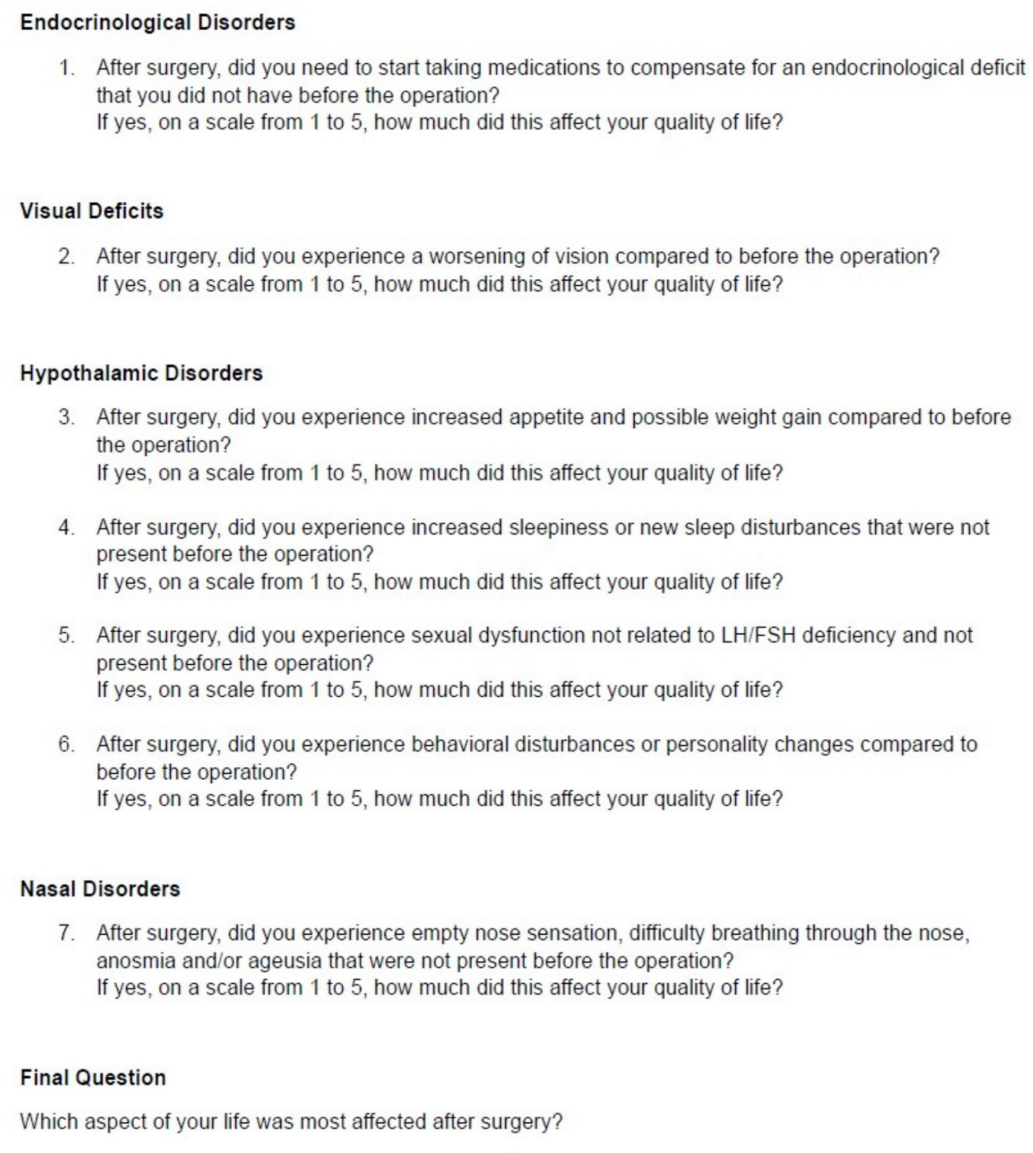
Questionnaire developed by the authors and administered by telephone to the patient cohort

The survey was administered by telephone and explored the following postoperative domains: need for HRT, visual disturbances, hypothalamic dysfunction (including alterations in appetite, sleep, sexual function, and personality), nasal breathing difficulties, ageusia and/or anosmia. For each domain, the impact on QoL was rated on a 5-point numerical scale, and patients were also asked to indicate the factor with the greatest overall impact. The questionnaire items are shown in Fig. 1.

### Statistical analysis

Descriptive statistics were reported as the mean and standard deviation for continuous variables and as frequency and percentage for nominal variables. The Shapiro–Wilk test was performed to assess the normality of the data distribution. Levene's test was used to evaluate the equality of variances. Differences between two groups were assessed using the Mann–Whitney U test or the Student's t-test for independent samples, depending on the assumptions met. Associations between nominal variables were evaluated using the chi-square (χ^2^) test as a univariate analysis. Subsequently, a multivariate analysis model was constructed using binary logistic regression, considering the variables that were statistically significant in the univariate analysis. A p-value less than < 0.05 was considered statistically significant. Statistical analyses were performed using SPSS software (version 25.0) and Jamovi software (Jamovi project [2019]. Jamovi [version 2.3.26.0] [computer software]; https://www.jamovi.org).

## Results

A total of 35 patients were included (14 females, 40%; 21 males, 60%), with a mean age of 54.5 years (range 28–75). Five patients were excluded because they were treated using a transventricular (3/5) or transcranial approach (2/5). Based on this, patients were divided into two groups: Group 1 (< 55 years, n = 17; 48.6%) and Group 2 (> 55 years, n = 18; 51.4%). Preoperatively, 20 patients (57.1%) had a BMI < 30, and 15 (42.9%) had a BMI > 30. At the time of surgery, 22 (62.9%) were non-smokers, while 13 (37.1%) were smokers. From an endocrinological standpoint, 11 patients (31.4%) presented with panhypopituitarism, and 24 (68.6%) had either isolated hormonal deficits or preserved pituitary function. Diabetes insipidus was present in 8 patients (22.9%). Visual deficits were observed in 24 patients (68.6%). Histologically, 19 cases (54.3%) were adamantinomatous and 16 (45.7%) papillary craniopharyngiomas, according to World Health Organization (WHO) 2021 classification.

Postoperative complications occurred in 14 patients (40%). Specifically, 7 had CSF leaks, 5 experienced hemorrhage, 4 developed pneumocephalus, and 5 had infections (2 meningitis, 2 pneumonia, 1 abdominal wound infection). Endocrine function improved or remained stable in 17 patients (48.6%), while 18 (51.4%) showed postoperative worsening. Visual function improved in 15 patients (42.9%), whereas 20 patients (57.1%) had stable or worsened deficits. Only one patient experienced a mild subjective worsening of overall visual acuity; therefore, stable and worsened cases were grouped into a single category.

During follow-up, recurrence or residual tumor growth occurred in 9 patients (25.7%). Among them, 8 (22.9%) received adjuvant radiotherapy, and 1 (2.9%) underwent reoperation. The mean follow-up duration was 82 months (median: 91.5 months; range: 12–156 months).

Detailed clinical and demographic data are summarized in Table 1.

**Table 1:** Descriptive results

	No. of patients	%
**Sex**	F:M 14:21	40%:60%
**Age (mean value)**	54.5 (range 28—75)	
Group 1 (< 55 years)	17	48.6%
Group 2 (> 55 years)	18	51.4%
**Preoperative BMI**		
BMI > 30	15	42.9%
BMI < 30	20	57.1%
**Smoke**	Y:N 13:22	37.1%:62.9%
**Preoperative endocrine function**		
Anterior panhypopituitarism	11	31.4%
Diabetes insipidus	8	22.9%
**Preoperative visual deficit**	24	68.6%
**Histopathology**		
Adamantinomatous	19	54.3%
Papillary	16	45.7%
**Surgical technique**		
EEA	35	100%
Preoperative LD	27	77.1%
Preoperative EVD	2	5.7%
Fat graft	35	100%
Hadad-Bassagasteguy flap	35	100%
Pituitary stalk resected	20	57.1%
Pituitary stalk preserved	15	42.9%
GTR	26	74.3%
STR	9	25.7%
**Postoperative Complications**	14	40%
CSF leak	7/14	50%
Hemorrhage	5/14	35.7%
Pneumocephalus	4/14	28.6%
Infection	5/14	35.7%
**Postoperative endocrine function**		
Improvement/Stability	17	48.6%
Worsening	18	51.4%
**Postoperative visual function**		
Improvement	15	42.9%
Worsening/Stability	20	57.1%
**Recurrence/Residual growth**	9	25.7%
**Adjuvant radiotherapy**	8	22.9%
**Surgical reintervention**	1	2.9%

### Surgical evaluation

All patients underwent surgery via an extended EEA. A preoperative lumbar drain (LD) was placed in 27 of 35 patients (77.1%) and typically removed on POD-2, barring complications. In 2 patients (5.7%), an external ventricular drain (EVD) was urgently placed due to acute hydrocephalus. In all cases, a paraumbilical autologous fat graft and a pedicled nasoseptal flap were used for multilayer skull base reconstruction. The pituitary stalk was sectioned in 20 patients (57.1%) and preserved in 15 (42.9%). GTR was achieved in 26 cases (74.3%), while 9 patients (25.7%) underwent subtotal resection (STR).

Surgical details are summarized in Table 1.

### Quality of life assessment

The responses to the QoL questionnaire are summarized in Table 2. In the “Worst” column, patients indicated the factor with the greatest impact on their QoL. The most frequently reported were weight gain (9/35, 25.7%) and sexual dysfunction (7/35, 20%), followed by the need for HRT and visual deficits (6/35 each, 17.1%). Nasal breathing difficulties were identified by 5 patients (14.3%), while sleep and behavioral disturbances were each reported by 1 patient (2.9%).

**Table 2:** Quality of life questionnaire results

Patient	HRT	Vision	Weight	Sleep	Sexuality	Behavior	Nose	Worst	Total
1	3	4	0	2	5	3	2	Sexuality	22
2	2	0	5	4	4	4	4	Weight	25
3	4	1	4	1	1	4	5	Nose	25
4	1	0	2	0	5	5	5	Nose	18
5	1	2	1	1	0	2	0	Vision	8
6	5	3	1	1	1	3	5	Nose	21
7	1	5	2	3	4	1	5	Nose	26
8	2	0	1	0	2	0	0	HRT	5
9	1	2	5	4	5	0	0	Weight	18
10	4	1	4	0	2	3	0	HRT	18
11	3	2	2	4	0	0	4	Sleep	20
12	2	0	5	2	4	1	0	Weight	14
13	5	0	5	1	0	5	1	Weight	22
14	3	0	0	2	4	0	0	HRT	9
15	2	2	3	5	5	0	1	Sexuality	23
16	5	2	4	4	4	1	0	HRT	21
17	5	5	5	5	5	5	5	Behavior	40
18	1	2	1	4	4	0	2	Sexuality	19
19	5	1	5	4	0	0	5	Weight	24
20	5	5	0	0	0	0	4	Vision	19
21	5	0	4	3	5	1	4	HRT	30
22	4	5	4	4	4	4	0	Vision	29
23	0	4	0	3	0	0	0	Vision	9
24	3	0	3	3	3	0	0	Sexuality	12
25	4	5	2	0	0	0	5	Nose	16
26	5	5	5	4	5	4	0	Sexuality	35
27	5	4	5	4	5	3	4	Weight	33
28	5	5	0	4	0	0	0	Vision	18
29	0	0	4	4	5	0	0	Weight	13
30	1	0	0	0	0	0	0	HRT	1
31	3	0	0	0	3	1	2	Sexuality	10
32	5	5	5	5	5	0	0	Vision	25
33	4	1	5	0	5	0	5	Weight	22
34	3	0	3	4	4	3	3	Sexuality	22
35	3	0	5	2	4	0	2	Weight	16

### Statistical analysis

Our analysis revealed several statistically significant findings regarding QoL in patients undergoing EEA for craniopharyngioma. Patients over 55 years reported a greater impact on QoL related to HRT compared to younger patients, with mean scores of 3.67 ± 1.68 vs. 2.59 ± 1.50 (p = 0.041). A higher perceived burden was also observed in patients without preoperative anterior panhypopituitarism compared to those with existing deficits (3.54 ± 1.61 vs. 2.27 ± 1.49; p = 0.033), as well as in those who experienced postoperative endocrine worsening (3.72 ± 1.36 vs. 2.53 ± 1.77; p = 0.041). Moreover, patients with preoperative visual deficits reported greater QoL impairment than those without (3.58 ± 1.81 vs. 2.18 ± 1.83; p = 0.032).

Patients with stable or worsened visual deficit after surgery reported higher QoL impact scores (2.85 ± 1.98) compared to those who experienced visual improvement (0.93 ± 1.58; p = 0.005), with females reporting greater discomfort than males (3.07 ± 1.90 vs. 1.33 ± 1.85; p = 0.012).

Postoperative weight gain had a greater impact in patients with a preoperative BMI > 30 (3.73 ± 1.49 vs. 2.20 ± 2.07; p = 0.039).

Sleep disturbances were more frequent in patients with adamantinomatous craniopharyngiomas compared to the papillary subtype (3.11 ± 1.52 vs. 1.75 ± 1.81; p = 0.026).

Although sexual dysfunction did not reach statistical significance overall, it was more frequently reported by males (3.33 ± 1.80 vs. 2.36 ± 2.34), older patients (3.28 ± 1.99 vs. 2.59 ± 2.12), and individuals who underwent pituitary stalk resection (3.50 ± 1.96 vs. 2.20 ± 2.01). Higher scores were also observed in patients with worsened postoperative endocrine function (3.17 ± 2.18) and those who underwent GTR (3.23 ± 1.92 vs. 2.11 ± 2.32).

Behavioral changes were more commonly reported in older patients (2.17 ± 1.72 vs. 0.82 ± 1.70; p = 0.010), while nasal breathing difficulties were more frequent among smokers (3.23 ± 2.05 vs. 1.41 ± 1.92; p = 0.023).

### Pituitary stalk resection

Pituitary stalk resection was significantly associated with a higher rate of GTR (18 vs. 8; p = 0.014 univariate, p = 0.045 multivariate), but also with worse postoperative endocrine outcomes (14 vs. 6; p = 0.011 univariate, p = 0.031 multivariate).

The “hypothalamic-pituitary function” score integrates the burden of endocrine therapy, weight issues, sexual dysfunction, sleep disorders, and behavioral changes. This was significantly higher in patients over 55 (15.1 ± 5.31 vs. 10.7 ± 5.53; p = 0.048) and in those who underwent stalk resection (14.8 ± 5.79 vs. 10.5 ± 5.44; p = 0.034).

Full statistical results are detailed in Table 3 and 4.

**Table 3:** Statistically significant results

Outcome variable	Independent variable	Comparison	No. of patients	Mean ± SD	*p*-value
HRT	Age	< 55 yrs vs > 55 yrs	17 vs 18	2.59 ± 1.50 vs 3.67 ± 1.68	**0.041**
	Preop. anterior panhypopituitarism	No vs Yes	24 vs 11	3.54 ± 1.61 vs 2.27 ± 1.49	**0.033**
	Preop. visual deficit	No vs Yes	11 vs 24	2.18 ± 1.83 vs 3.58 ± 1.81	**0.032**
	Postop. endocrine function	Improvement/Stability vs Worsening	17 vs 18	2.53 ± 1.77 vs 3.72 ± 1.36	**0.041**
Visual deficit	Sex	Female vs Male	14 vs 21	3.07 ± 1.90 vs 1.33 ± 1.85	**0.012**
	Postop. visual function	Improvement vs Worsening/Stability	15 vs 20	0.93 ± 1.58 vs 2.85 ± 1.98	**0.005**
Weight gain	BMI	< 30 vs > 30	20 vs 15	2.20 ± 2.07 vs 3.73 ± 1.49	**0.039**
Sleep disorders	Histopathology	Adamantinomatous vs Papillary	19 vs 16	3.11 ± 1.52 vs 1.75 ± 1.81	**0.026**
Behavioral disorders	Age	< 55 yrs vs > 55 yrs	17 vs 18	0.82 ± 1.70 vs 2.17 ± 1.72	**0.010**
Nasal disorders	Smoking	No vs Yes	22 vs 13	1.41 ± 1.92 vs 3.23 ± 2.05	**0.023**
Hypothalamic–pituitary function	Age	< 55 yrs vs > 55 yrs	17 vs 18	10.7 ± 5.5 vs 15.1 ± 5.3	**0.048**
	Pituitary stalk	Preserved vs Resected	15 vs 20	10.5 ± 5.4 vs 14.8 ± 5.8	**0.034**

**Table 4:** Statistically significant results of pituitary stalk management analysis

Variable		Pituitary stalk		*p*-value (univ.)	*p*-value (multi.)
	Total	Preserved	Resected		
**EOR**				**0.014**	**0.045**
STR	9	7	2		
GTR	26	8	18		
**Postoperative endocrine function**				**0.011**	**0.031**
Improvement/Stability	17	11	6		
Worsening	18	4	14		

## Discussion

Craniopharyngiomas are WHO grade I tumors [19]. Despite their benign histopathology, their proximity to critical structures, such as pituitary gland, optic chiasm, hypothalamus, and third ventricle, often results in significant neurological and endocrinological complications, both from the tumor itself and from its treatment. As these patients typically require lifelong medical care and monitoring, identifying modifiable factors that impact postoperative QoL is of paramount importance [20].

While the impact of craniopharyngioma on QoL has been well documented in pediatric populations [4, 23], data on adult patients remain scarce. In 2021, Lucia et al. evaluated QoL in a cohort of 20 adults, highlighting that over half were unable to resume their preoperative job functions. However, 78% maintained social independence in daily life [20]. Notably, this study did not investigate preoperative factors that might predict postoperative QoL. Another recent study conducted by Mineni et al. proposed an additional scoring system for postoperative QoL assessment. However, unlike our study, it did not specifically focus on patients undergoing endoscopic endonasal surgery, nor did it evaluate the relationship between QoL outcomes and pituitary stalk sectioning. [26] To address this gap, we conducted a study assessing long-term QoL in adult craniopharyngioma patients, with a focus on identifying clinical and preoperative predictors of postoperative outcomes.

We administered a custom-designed questionnaire (Fig. 1) to 35 patients who had undergone surgery at least 12 months earlier, allowing for evaluation of long-term effects. The questionnaire addressed four main domains: (1) need for postoperative HRT; (2) visual deficits; (3) hypothalamic disturbances, including weight gain, sleep disorders, sexual dysfunction, and personality changes; and (4) nasal complications, including nasal breathing difficulty, ageusia, and anosmia.

The following sections analyze the statistical correlations between questionnaire responses and clinical data to identify potential preoperative predictors of long-term QoL.

### Hormone replacement therapy

The need for postoperative HRT is a key factor affecting QoL in patients with craniopharyngioma. Central diabetes insipidus, a common postoperative complication, has been identified as a risk factor for depressive symptoms [18]. Although Desmopressin is generally effective in managing polyuria, its impact on overall QoL remains inconsistent, with many patients reporting ongoing challenges despite adequate symptom control [14, 28].

**Fig. 2:**
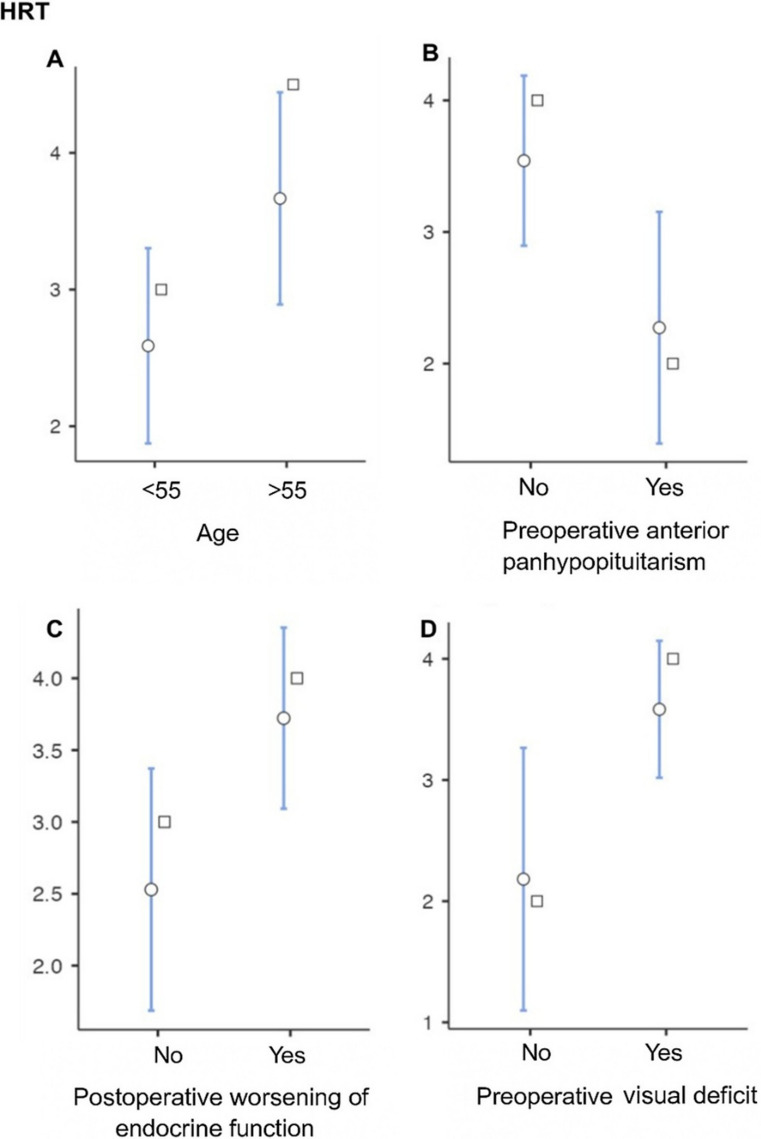
Statistical analysis of HRT in relation to (**A**) age (*p* = 0.041), (**B**) preoperative anterior panhypopituitarism (*p* = 0.033), (**C**) postoperative worsening of endocrine function (*p* = 0.041), and (**D**) presence of preoperative visual deficits (*p* = 0.032). The circle indicates the mean, and the square indicates the median

Our questionnaire specifically assessed the requirement for new endocrine therapies introduced after surgery and asked patients to rate their impact on QoL (scale 1–5). As shown in Table 2, patients requiring multiple hormone replacements reported significantly lower QoL. This is particularly relevant in adult and elderly populations, where adherence to complex medication regimens can be problematic [13]. Our findings suggest that patients with no or only partial endocrine deficits preoperatively experienced a greater decline in QoL post-surgery when faced with the burden of polypharmacy (Fig. 2).

Additionally, patients with preoperative visual deficits tended to report lower QoL scores. These individuals often show limited postoperative improvement or persistent visual impairment, and when combined with the demands of HRT, their overall burden increases, further diminishing QoL (Fig. 2).

### Visual impairment

Visual impairment is a major determinant of reduced QoL in patients with craniopharyngioma, with an impact closely related to the severity of the deficit. Visual loss compromises autonomy, limits daily activities, and imposes a substantial psychological burden, often leading to frustration, reduced self-efficacy, and impaired social and occupational functioning [38].

Given that visual field deficits are among the main indications for surgical intervention, both in craniopharyngiomas and other sellar lesions such as pituitary adenomas, we evaluated postoperative visual outcomes in our cohort. Patients were asked whether they experienced new or worsened visual deficits following surgery and to rate the impact on their QoL (scale 1–5). As shown in Table 3, our findings are consistent with previous studies identifying visual impairment as a key predictor of poor QoL in this population, alongside hormonal dysfunction and elevated BMI [7].

**Fig. 3:**
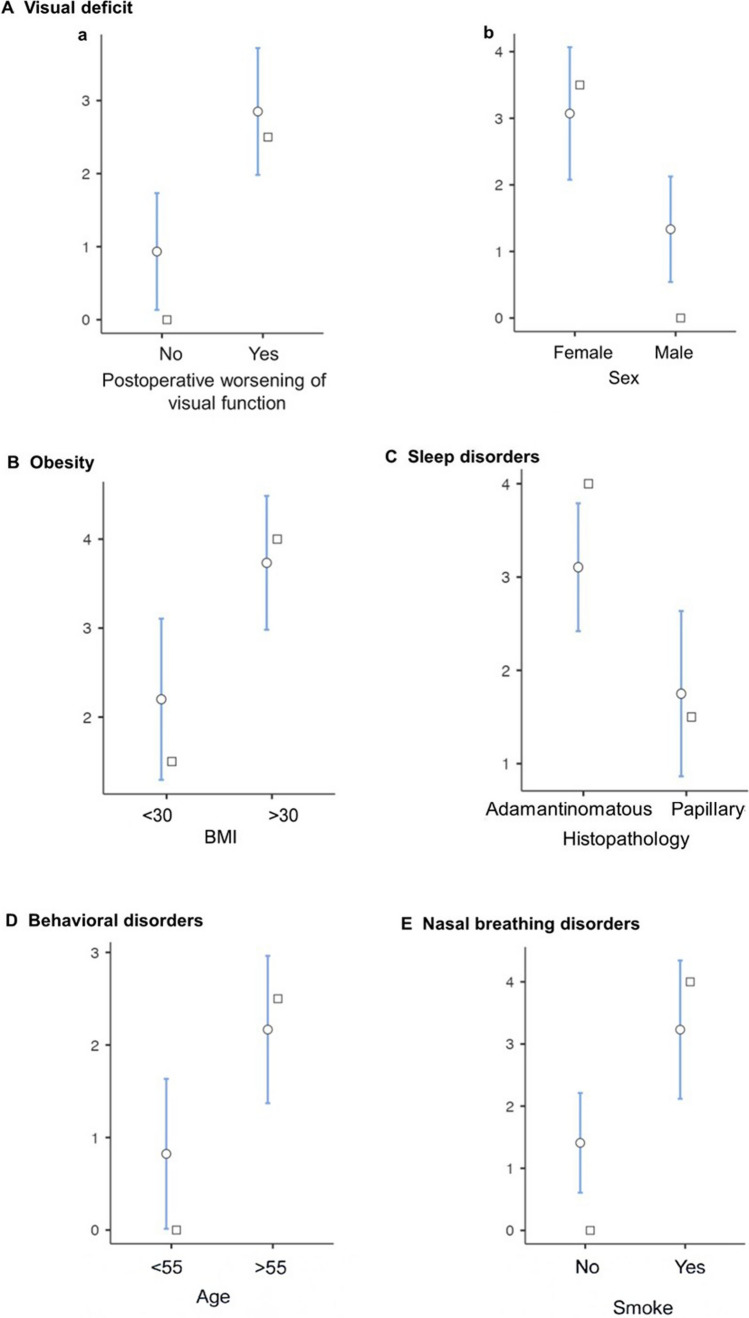
Statistical analysis of (**A**) visual deficit in relation to (a) postoperative worsening of visual function (*p* = 0.005) and (b) sex (*p* = 0.012); (**B**) obesity in relation to BMI (*p* = 0.039); (**C**) sleep disorders in relation to histopathology (*p* = 0.026). (**D**) behavioral disorders in relation to age (*p* = 0.010); (**E**) nasal breathing disorders in relation to smoking (*p* = 0.023). The circle indicates the mean, and the square indicates the median

Dekkers et al. demonstrated that visual field deficits are the strongest independent predictor of low QoL in adults with craniopharyngioma, correlating with depression, fatigue, and reduced motivation [11]. Similarly, Kawamata et al. reported that visual disturbances significantly lower functional performance postoperatively, as measured by the Karnofsky Performance Scale (KPS) and health-related QoL (HRQoL) indices [16] (Fig. 3).

Interestingly, in our cohort, visual impairment had a more pronounced negative effect on QoL in female patients. Although female sex is not a disease-specific predictor, it has been associated with lower QoL scores across multiple domains, including physical functioning, pain, fatigue, and social limitations [7, 11, 31]. While a direct link between visual impairment and sex-specific QoL differences has not been firmly established, our data suggest that women may experience greater psychosocial difficulty adapting to physical deficits (Fig. 3A).

### Obesity

Hypothalamic obesity significantly impairs long-term QoL in patients with craniopharyngioma, affecting both psychosocial well-being and metabolic health. Common long-term consequences include daytime sleepiness, fatigue, circadian rhythm disruption, gastrointestinal and pulmonary symptoms (e.g., diarrhea, dyspnea), memory deficits, and neuropsychological disturbances [25, 42]. In our questionnaire, patients were asked whether they had experienced increased appetite and/or weight gain after surgery, and to rate its impact on their QoL on a 5-point scale. Our findings suggest that patients with a high preoperative BMI may already suffer from the effects of hypothalamic obesity. For these individuals, additional weight gain can be particularly distressing, intensifying psychosocial issues such as anxiety, depression, disordered eating, and low self-esteem. Furthermore, weight gain compounds the physical complications of obesity, including diabetes, dyslipidemia, cardiovascular disease, ischemic stroke, and greater infection risk. Together, these factors significantly contribute to reduced QoL (Fig. 3B).

### Sleep disorders

Sleep disturbances are a major concern in patients with craniopharyngioma. These issues may arise from tumor-related damage to structures regulating the sleep–wake cycle, iatrogenic injury, or as a consequence of hypothalamic obesity. Hypothalamic dysfunction, particularly involving the suprachiasmatic nucleus, is central to the pathogenesis of sleep disorders [33].

In our cohort, patients with adamantinomatous craniopharyngioma reported more pronounced sleep disturbances than those with papillary tumors. This is difficult to interpret, as no current evidence supports a higher prevalence of hypothalamic dysfunction in either histological subtype (Fig. 3C).

However, up to 35% of patients with adamantinomatous craniopharyngioma exhibit signs of hypothalamic dysfunction, including sleep disruption, at diagnosis [21]. Postoperatively, hypothalamic dysfunction remains one of the main comorbidities in this group. Anatomically, adamantinomatous tumors often involve the sellar and suprasellar regions, frequently affecting the pituitary stalk, while papillary craniopharyngiomas typically arise in the third ventricle, often sparing the stalk [30].

This anatomical distinction may partly explain the higher rate of postoperative hypothalamic disturbances in adamantinomatous cases. Still, the significance of this finding could require further investigation.

### Sexual dysfunction

Sexuality is a complex domain influenced by neural, endocrinological, and genetic factors. Key neural structures involved include the limbic system—hypothalamus, amygdala, hippocampus, and septal nuclei—that regulate motivation and emotional processing. Notably, the medial preoptic area of the anterior hypothalamus integrates inputs to trigger sexual motivation [6]. Craniopharyngioma growth can damage the hypothalamus, pituitary gland, and stalk, causing gonadotropin deficiency and adult hypogonadism, which often results in sexual dysfunction and reduced libido [34, 44]. Additionally, GH deficiency has been linked to high rates of sexual dysfunction in both sexes [22]. Sexual well-being is a critical component of QoL, as sexual disorders may lower self-esteem and impair intimate relationships [7]. These changes affect not only the patient but also their social support network, underscoring the need to consider psychosocial factors in managing chronic illness [6].

### Behavioral disorders

Hypothalamic damage can impair functions such as feeding, sleep, reproduction, and thermoregulation. Due to its connections with the limbic system, emotional regulation is often affected. Psychiatric disorders frequently accompany changes in endocrine function, eating behavior, sleep, and autonomic regulation [1]. In the literature, many case reports describe craniopharyngiomas presenting with altered mental status, including rapidly progressive dementia, mania, catatonia, and psychosis [5, 37]. The high prevalence of socio-behavioral and emotional dysfunctions in these patients is likely multifactorial, involving frontal lobe dysfunction, hormonal imbalances, and hypothalamic/diencephalic impairment [43]. Additionally, oxytocin, which has been recognized as playing a fundamental role in social functioning, may be reduced in patients with hypopituitarism [9, 10].

Our findings show that behavioral and personality changes occur more frequently in older patients (Fig. 3D). This may be explained by the influence of physical and social factors together with the cumulative impact of past experiences and aging-specific stressors on mental health in advanced age. Older adults face higher risks of depression and anxiety due to health decline, poor living conditions, and limited support [39]. Furthermore, fear of tumor recurrence and treatment-related anxiety often increase with age, as autonomy decreases and social isolation rises [43]. Thus, both hypothalamic dysfunction and age-related psychosocial distress likely contribute to the greater impact of behavioral disorders on QoL in patients over 55.

### Nasal breathing disorders

The transsphenoidal approach for sellar and suprasellar lesions has been used in neurosurgery for over a century; however, the endonasal morbidity associated with this technique has only recently received scientific attention [27]. The nasal-sinus quality of life questionnaire, SNOT-22, was developed to assess symptoms in patients undergoing EEA [2].

In our questionnaire, patients reported symptoms such as nasal emptiness, nasal breathing difficulty, and taste or smell disturbances post-surgery, rating their impact on quality of life from 1 to 5. Our findings (Fig. 3E) align with a 2020 study by Shay et al., which showed that smokers undergoing EEA with nasoseptal flap reconstruction had worse total SNOT-22 scores compared to non-smokers [36]. Since all patients in our cohort had nasoseptal flap reconstruction, these results are consistent with existing literature. Notably, the study also found that smoking does not affect flap healing time [36].

### Pituitary stalk section role in patient quality of life

The management of craniopharyngiomas remains debated. Some advocate a conservative strategy of STR followed by radiotherapy to preserve pituitary function and minimize hypothalamic damage, especially in pediatric patients vulnerable to GH deficiency, obesity, and cognitive deficits [12]. This approach is based on the premise that STR combined with radiotherapy achieves tumor control rates comparable to GTR but with less morbidity. However, recurrence occurs in over one-third of cases with this strategy [35, 41]. Many studies comparing STR plus radiotherapy to GTR used craniotomy surgical approaches, which pose challenges in achieving GTR without significant comorbidities [35]. A 2021 national cohort study of 11.166 patients reported higher mortality and comorbidities after craniotomy GTR versus EEA [15].

**Fig. 4:**
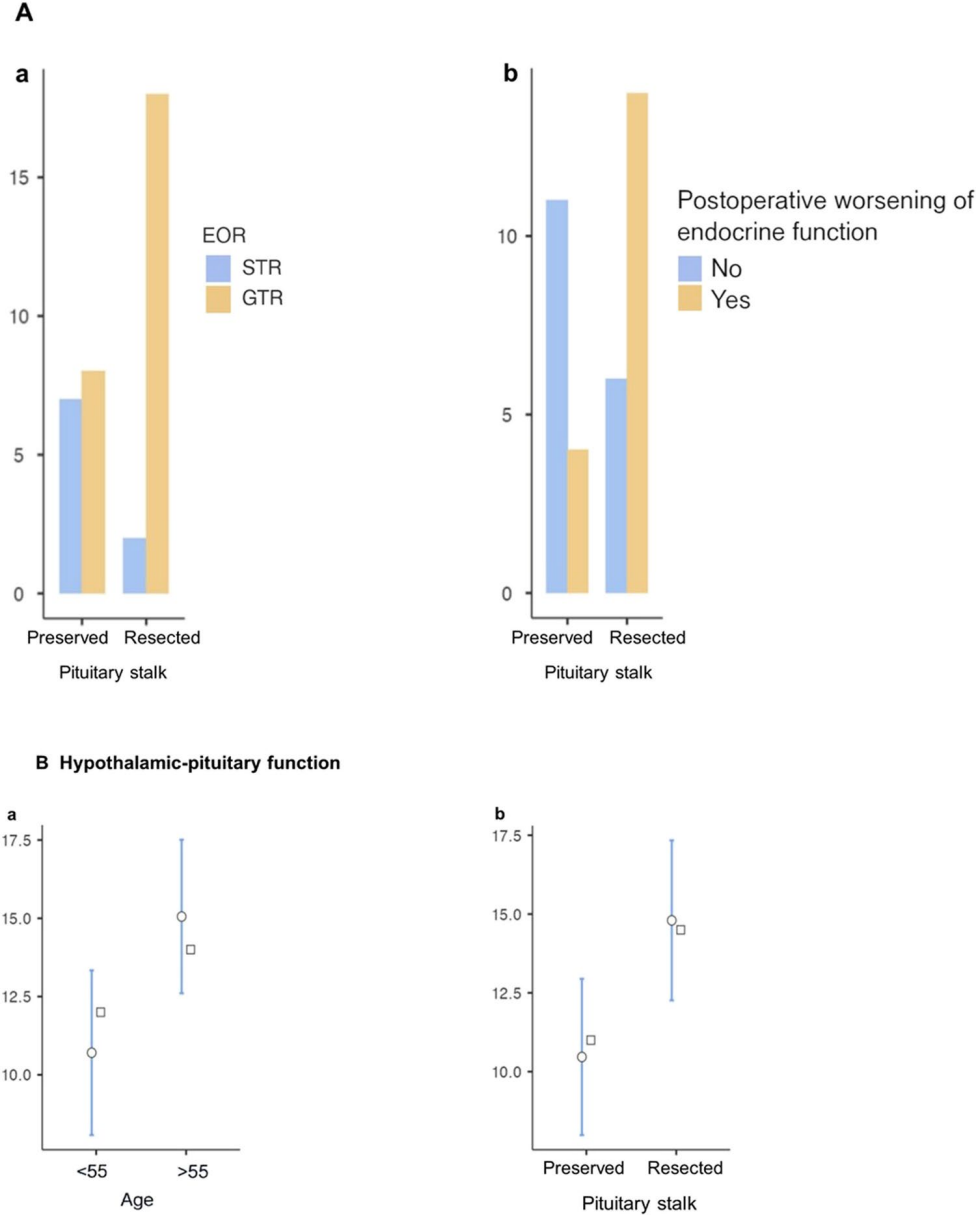
**A** Association between pituitary stalk management and (**a**) EOR (univariate *p* = 0.014, multivariate *p* = 0.045) and (**b**) postoperative worsening of endocrine function (univariate *p* = 0.011, multivariate *p* = 0.031). (**B**) Association between hypothalamic–pituitary function and (**a**) age (*p* = 0.048) and (**b**) pituitary stalk management (*p* = 0.034). The circle indicates the mean, and the square indicates the median

With advances in endoscopic techniques, multiple studies have shown that GTR provides superior long-term tumor control, overall survival, and disease-free survival compared to STR with radiotherapy [3, 8, 29]. Preservation of the pituitary stalk, while reducing postoperative panhypopituitarism and diabetes insipidus, may increase recurrence risk due to microscopic residual tumor cells. This highlights the critical role of EOR in craniopharyngioma management. Nevertheless, pituitary stalk preservation is associated with lower rates of postoperative panhypopituitarism and diabetes insipidus [3] (Fig. 4A).

Our study specifically examined the impact of pituitary stalk section on long-term HRQoL in adults undergoing EEA. Given the balance between aggressive resection and preserving pituitary function, we assessed how stalk resection influences QoL. We grouped questionnaire items related to hormone replacement, weight, sexual function, sleep, and behavior into a “hypothalamic-pituitary functions” category. Analysis showed that stalk section worsens QoL, particularly through increased endocrinological therapy needs and hypothalamic disturbances. These dysfunctions had a greater negative effect in patients older than 55 years and those with stalk resection (Fig. 4B).

Therefore, in older patients, careful consideration is needed to balance resection extent with QoL implications. Preoperative counseling should involve patients in shared decision-making regarding the risks and benefits of stalk preservation versus resection to optimize surgical goals and postoperative QoL.

The retrospective design and the limited sample size represent the main limitations of this study. Moreover, both the patient-administered questionnaire and the assessment of QoL are inherently subjective, varying across individuals and therefore not consistently providing objective or unequivocal data. Nevertheless, to our knowledge, this is the first attempt to investigate the correlation between long-term QoL and pituitary stalk section in adult patients with craniopharyngioma treated via EEA.

## Conclusions

This study assessed long-HRQoL in adults undergoing EEA for craniopharyngioma, highlighting a significant correlation between QoL and pituitary stalk resection. While stalk section facilitates GTR, it substantially worsens endocrine function, leading to impaired QoL, especially in patients older than 55 years. These findings emphasize the need to carefully balance the benefits and risks of pituitary stalk resection during surgery. Future multicenter, prospective studies are essential to provide further insights into this field and contribute to the development of management guidelines for the surgical treatment of these lesions.

## Data Availability

All data generated or analyzed during this study are included in this published article (and its supplementary information files).

## Abbreviations

QoL: Quality of life
CSF: Cerebrospinal fluid
EOR: Extent of resection
GTR: Gross total resection
EEA: Endoscopic endonasal approach
CTA: CT angiography
HRT: Hormone replacement therapy
POD: Postoperative day
WHO: World Health Organization
LD: Lumbar drain
EVD: External ventricular drain
STR: Subtotal resection
GH: Growth hormone
KPS: Karnofsky Performance Scale
HRQoL: Health-related QoL
